# Human Gene Functional Network-Informed Prediction of HIV-1 Host Dependency Factors

**DOI:** 10.1128/mSystems.00960-20

**Published:** 2020-11-03

**Authors:** Chen Fu, Shiping Yang, Xiaodi Yang, Xianyi Lian, Yan Huang, Xiaobao Dong, Ziding Zhang

**Affiliations:** a State Key Laboratory of Agrobiotechnology, College of Biological Sciences, China Agricultural University, Beijing, China; b Department of Genetics, School of Basic Medical Sciences, Tianjin Medical University, Tianjin, China; University of Vienna

**Keywords:** HIV-1, host dependency factors, prediction, machine learning, gene functional network

## Abstract

Human immunodeficiency virus type 1 (HIV-1) depends on a class of host proteins called host dependency factors (HDFs) to facilitate its infection. So far experimental efforts have detected a certain number of HDFs, but the gene inventory of HIV-1 HDFs remains incomplete. Here, we implemented an existing network-based gene discovery strategy to predict HIV-1 HDFs. First, an encoding scheme based on a publicly available human tissue-specific gene functional network (GIANT; http://giant.princeton.edu/) was designed to convert each human gene into a 25,825-dimensional feature vector. Then, a random forest-based predictive model was trained on a data set containing 868 known HDFs and 1,736 non-HDFs. Through 5-fold cross-validation, an independent test, and comparison with one existing method, the proposed prediction method consistently revealed accurate and competitive performance. The highlight of our method should be ascribed to the introduction of the GIANT encoding scheme, which contains rich information regarding gene interactions. By merging known HDFs and genome-wide HDF prediction results, network analysis was conducted to catch the common patterns of HDFs in the context of the GIANT network. Interestingly, HDFs reveal significantly lower betweenness than HIV-1-interacting human proteins (i.e., HIV targets). In the meantime, the functional roles of HDFs were also examined by mapping all the HDF candidates into human protein complexes. Especially, we observed the frequent co-occurrence of HDFs and HIV targets at the protein complex level. Collectively, we hope the proposed prediction method not only can accelerate the HDF identification and antiviral drug target discovery, but also can provide some mechanistic insights into human-virus relationships.

**IMPORTANCE** Identification of HIV-1 HDFs remains a crucial step to understand the complicated relationships between human and HIV-1. To complement the experimental identification of HDFs, we have implemented an existing network-based gene discovery strategy to predict HDFs from the human genome. The core idea of the proposed method is that the rich information deposited in host gene functional networks can be effectively utilized to infer the potential HDFs. We hope the proposed prediction method could further guide hypothesis-driven experimental efforts to interrogate human–HIV-1 relationships and provide new hints for the development of antiviral drugs to combat HIV-1 infection.

## Abstract (precis)

Identification of HIV-1 HDFs remains a crucial step to understand the complicated relationships between human and HIV-1. To complement the experimental identification of HDFs, we have implemented an existing network-based gene discovery strategy to predict HDFs from the human genome. The core idea of the proposed method is that the rich information deposited in host gene functional networks can be effectively utilized to infer the potential HDFs. We hope the proposed prediction method could further guide hypothesis-driven experimental efforts to interrogate human–HIV-1 relationships and provide new hints for the development of antiviral drugs to combat HIV-1 infection.

## INTRODUCTION

As a kind of obligate intracellular pathogens, viruses contain small genomes that encode a limited number of proteins. To carry out their activities in host cells, viruses need to exploit host proteins for entry, replication, and transmission. In general, such host proteins are referred to as host dependency factors (HDFs) (1, 2). Biologically, the characterization of HDFs remains an important step to decipher human-virus relationships (3). In the meantime, HDFs can serve as potential antiviral drug targets. Indeed, such a target discovery strategy is increasingly attractive, since it can effectively avoid drug resistance in comparison to therapeutic targeting of viral proteins (4–6).

As an infectious virus, HIV-1 continuously poses a serious threat to human health. Mechanistic understanding of human–HIV-1 interaction has been of long-term research interest to the community. The genome of HIV-1 encodes only 19 proteins. Consequently, it has to rely on HDFs to complete its life cycle (7). In the past decades, many experimental methods, such as small interfering RNA (siRNA)-based screens (8) and CRISPR/Cas9-based screens (9, 10), have been explored to identify HIV-1 HDFs (11–15). Regarding siRNA-based screens, individual human genes are first knocked down through RNA interference, then the effects of viral infection (e.g., levels of viral protein expression or production of viral particles in human cells) are measured to find potential HDFs. As a novel and powerful loss-of-function technique, CRISPR/Cas9 has also been applied to the detection of HDFs with higher sensitivity and specificity (10). Until now, the experimentally identified HIV-1 HDFs have provided further insights into the functional roles of HIV-1 HDFs. Moreover, the relationship between HDFs and HIV-1-interacting human proteins (i.e., HIV targets) has been examined in the context of human protein-protein interaction (PPI) networks (16, 17). Regarding the antiviral drug discovery, HDF-targeted drugs have been successfully developed. For instance, one HIV-1 HDF called CCR5 could serve as a coreceptor for HIV-1 infection of CD4^+^ T cells and macrophages, and small molecule inhibitors of CCR5 have been developed as effective anti-HIV drugs (18).

Thanks to the development of experimental techniques, more and more HIV-1 HDFs have been continuously discovered, especially with the application of CRISPR/Cas9-based screens (2, 10). In the meantime, it has been reported that high false-negative rates exist in previous genome-wide siRNA-based HDF screens (19). The evidence above clearly indicates that the current catalogs of HIV-1 HDFs remain incomplete. Additionally, experimental methods are often time-consuming and laborious. In this regard, cost-effective computational methods may offer a promising alternative solution for complementing the experimental identification of HDFs. Indeed, the available HDF data have provided a solid foundation for the development of prediction methods. Considering the functional diversity of HDFs, conventional sequence information-based protein family prediction is not suitable for this task. Rather, network-based gene discovery (16, 20, 21) may provide an effective alternative solution to detect HDFs. Based on the hypothesis that the network topologies of known HDFs within human PPI networks can be employed to detect new HDFs, Murali et al. initially predicted HIV-1 HDFs through the introduction of a graph-theoretic approach called SinkSource (16). Recently, Ackerman et al. proposed a method of integrating human PPI networks with human-virus PPIs to detect HDFs of influenza viruses (21). In addition to successfully predicting novel HDFs, the topology relationships between HDFs and virus-interacting proteins in the context of human PPI networks have been characterized. The aforementioned prediction and analysis of HDFs indicated that the network-informed strategy is powerful for novel HDF discovery.

In comparison to pure PPI networks, genome-wide functional networks may be more comprehensive to represent the complex gene/protein associations within cellular systems. In 2015, Troyanskaya and coworkers developed a series of tissue-specific functional gene interaction networks through a Bayesian data integration strategy (22). The integrated data types include thousands of PPI, gene expression, and regulatory sequence data sets. Moreover, they have constructed a web server called GIANT (Genome-Scale Integrated Analysis of Networks in Tissues) to make the predicted functional gene interaction networks applicable to the community. Based on the GIANT network, for instance, Krishnan et al. employed a machine learning approach to conduct genome-wide prediction of autism risk genes. They successfully predicted hundreds of autism risk gene candidates with little or no prior genetic evidence, many of which have been experimentally validated (23).

Inspired by the successful applications of network-based gene discovery (22–25), in this work we implemented a GIANT network-informed prediction method of HIV-1 HDFs with the assistance of machine learning algorithms. We will elaborate the overall computational framework, methodology details, performance assessment, and comparison of the proposed HDF predictor. In the meantime, we will also report the comprehensive network and functional analyses of HDF candidates inferred from genome-wide prediction, which will allow us to better understand the global landscape of HIV-1 HDFs.

## RESULTS AND DISCUSSION

### The computational framework of the proposed network-informed HDF prediction.

**FIG 1:**
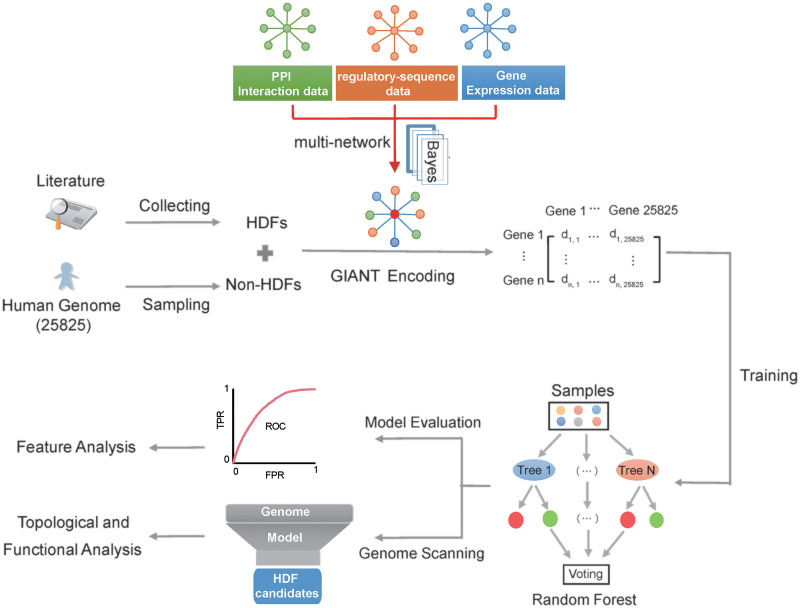
Flowchart of the proposed HIV-1 HDF prediction method. In the data set preparation step, we compiled positive genes (known HDFs) through literature searching and negative genes (i.e., non-HDFs) through random sampling of human proteins other than known HDFs. In the feature vector construction step, we employed the T-cell-specific GIANT network to convert each positive/negative sample into a 25,825-dimensional vector. In the model training and evaluation step, we introduced five popular machine learning methods, and RF was selected as the optimal machine learning algorithm through the 5-fold cross-validation and independent test. Moreover, the feature selection was conducted to rank the contributions of different features in the proposed encoding scheme. In the final step, we conducted genome-wide HDF screening based on the proposed method, and conducted topological analysis of the HDF candidates in the context of the GIANT network and examined the functional roles of HDF candidates in the context of human protein complexes.

The flowchart of the proposed prediction method is illustrated in Fig. 1. At first, we manually collected known HDFs with experimental evidence (i.e., positive samples) and selected non-HDFs (i.e., negative samples) through random sampling of human genes other than known HDFs. We further compiled them into a training data set covering 868 HDFs and 1,736 non-HDFs and an independent test set involving 276 HDFs and 552 non-HDFs. Then, the GIANT network was used to infer feature vectors for HDFs/non-HDFs. Based on the GIANT encoding scheme, five popular machine learning methods (i.e., random forest [RF], naive Bayesian [NB], *k*-nearest neighbors [KNN], logistic regression [LR], and support vector machine [SVM]) were adopted to build the corresponding predictive models. At last, a 5-fold cross-validation and an independent test were carried out to select the best predictive model. More details about the data set preparation, GIANT-based feature vector construction, machine learning algorithm implementation, and performance metrics are available in Materials and Methods.

### The performance of network-based HDF prediction.

**FIG 2:**
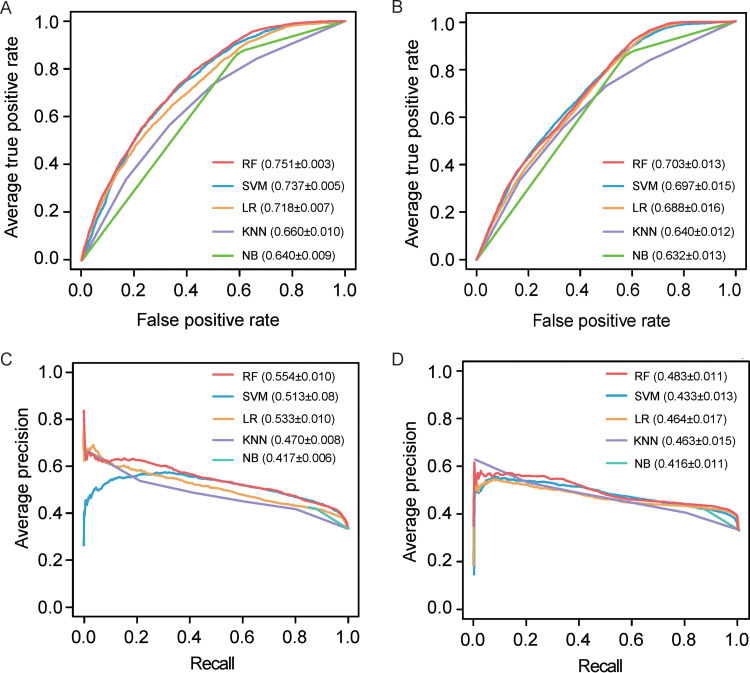
Performance comparison of prediction models based on different machine learning methods. (A) ROC curves of the 5-fold cross-validation. (B) ROC curves of the independent test. (C) PR curves of the 5-fold cross-validation. (D) PR curves of the independent test. Parameters in parentheses in panels A and B denote the AUC values of different models, while parameters in parentheses in panels C and D stand for the AUPRC values. Note that the AUC/AUPRC values are reported as average ± standard deviation (SD).

In this work, a 5-fold cross-validation and an independent test were carried out to stringently assess the model performance of different machine learning algorithms, which were first measured through the receiver operating characteristic (ROC) curve and the area under ROC curve (AUC). It should be noted that for fair comparison, the parameters in different algorithms were preliminarily optimized (i.e., the key parameters in each algorithm were optimized, while other parameters were set as default). As shown in Fig. 2A, the RF-based model performed the best (AUC = 0.751) in the 5-fold cross-validation, followed by SVM (AUC = 0.737), LR (AUC = 0.718), KNN (AUC = 0.660), and NB (AUC = 0.640). Figure 2B illustrates the ROC curves of the models in the independent test. Considering that the precision-recall (PR) curve is more suitable for characterizing the model performance with imbalanced positives and negatives, the PR curve and the area under PR curve (AUPRC) are also provided in Fig. 2C and D. Likewise, the RF-based model performed the best in either the 5-fold cross-validation or the independent test. For real application, it is important to quantify the performance at a low false-positive rate (FPR) control. At an FPR control of 10%, for instance, the corresponding sensitivity (with precision in parentheses) values for RF, SVM, LR, KNN, and NB are 31.2% (61.7%), 28.6% (57.1%), 29.2% (55.3%), 20.9% (54.2%), and 14.8% (43.2%) in the 5-fold cross-validation, respectively (Fig. 2A and C). Moreover, it is worth noting that the AUC/AUPRC values from the independent test revealed reasonably decreased performance in comparison to the 5-fold cross-validation, which should be ascribed to the fact that the positive samples in the training set and independent test set were selected from different experimental studies. Even so, different machine learning-based models showed the same performance rank in either the 5-fold cross-validation or the independent test, further suggesting the overall performance of these five machine learning-based models is robust.

Considering T cells are the principal targets for HIV-1 (26, 27), our predictive model is based on the T-cell-specific GIANT network. To demonstrate whether the predictive model is sensitive to different tissue-specific networks, we compared the performance of the T-cell-specific network against the networks of other tissues that are not known HIV-1 host cells. In terms of AUC or AUPRC, the T-cell-specific network slightly outperformed the epidermis tissue- and adipose tissue-specific networks (Table 1), implying the T-cell-specific network seems to have higher signal-to-noise ratio to some extent.

**TABLE 1:** Model performance based on different tissue-specific GIANT networks and PPI networks

Network type	5-fold cross-validation^*a*^		Independent test^*a*^	
	AUC	AUPRC	AUC	AUPRC
GIANT networks				
T cells	0.751 ± 0.003	0.554 ± 0.010	0.703 ± 0.013	0.483 ± 0.011
Adipose tissue	0.747 ± 0.002	0.546 ± 0.008	0.703 ± 0.015	0.476 ± 0.014
Epidermis tissue	0.747 ± 0.002	0.552 ± 0.006	0.701 ± 0.025	0.468 ± 0.022
PPI networks				
PPI network in this study^*b*^	0.643 ± 0.004	0.502 ± 0.011	0.552 ± 0.020	0.368 ± 0.025
InWeb_InBioMap^*b*^	0.669 ± 0.006	0.501 ± 0.010	0.590 ± 0.014	0.390 ± 0.012

a The results are based on five different repeats of negative sample selections, which are expressed as average ± SD.

b We used the same encoding strategy as the GIANT network to infer the compiled PPI network- or InWeb_InBioMap-based predictive model. Since there are a total of 16,745 proteins in the compiled PPI network, each sample can be converted into a 16,745-dimensional feature vector. Regarding the InWeb_InBioMap PPI network, the number of proteins is 16,948, and thus each sample can be represented as a 16,948-dimensional vector. To train and assess the compiled PPI network- or InWeb_InBioMap-based model, note that some HDFs in the original training and independent test sets were removed since they were not included in these two PPI networks.

We also reconstructed the proposed predictive model based on a human PPI network compiled in this study, which contains 344,703 interactions and 16,745 proteins. Rather than the GIANT network, the PPI network data used here are unweighted and tissue independent. To infer the PPI network-based encoding, the interaction score of an interacting protein pair was set to 1.0, whereas the interaction score of a noninteracting protein pair was set to 0.0. As shown in Table 1, the PPI network-based model achieved an AUC value of 0.643 (AUPRC = 0.502) in the 5-fold cross-validation and an AUC value of 0.552 (AUPRC = 0.368) in the independent test, which are much lower than those of the corresponding counterparts in the GIANT network-based predictive model. Likewise, we also retrained the RF model based on a systematically integrated PPI network called InWeb_InBioMap (28), which covers 580,075 interactions and 16,948 proteins in version 2016-09. In general, the InWeb_InBioMap model outperforms the model based on the PPI network compiled in this work, but it is still inferior to the GIANT network-based model (Table 1). Collectively, the above performance comparison of the GIANT network-based model and two PPI network-based models demonstrated that GIANT is a suitable gene network for HDF identification.

### Selection of different negative data sets.

It has been established that the real ratio of HDFs to non-HDFs is highly skewed, although the exact ratio of positives to negatives in the human genome remains elusive. To address this highly imbalanced classification task, the ratio of positives to negatives used in training/assessing machine learning models remains an open issue. On the one hand, models trained on balanced samples, as widely used in many classification tasks, cannot reflect reality. On the other hand, models trained on a highly imbalanced ratio will also inevitably generate biased results. In this context, a relatively imbalanced ratio of positives to negatives was often empirically adopted without strict optimization. Here, we conducted some computational analyses to investigate the different ratios of positives to negatives in model training and assessment. Supposing that the real ratio of HDFs to non-HDFs in the human proteome is 1:10, we trained predictive models based on four different ratios of HDFs to non-HDFs (1:1, 1:2, 1:5, and 1:10) and assessed the performance on an independent test set with a 1:10 ratio of HDFs to non-HDFs. By doing so, we can roughly examine the effects of different training sample ratios in the real application. Note that the HDFs in the training set and independent set were the same as those used in developing our original model. As shown in Table S1 in the supplemental material, the overall performance of RF-based models was only slightly affected by the sample ratios in training. Comparatively, the training set with a 1:2 or 1:5 ratio of HDFs to non-HDFs yielded better performance than the ratios of 1:1 and 1:10 (Table S1). Thus, the above analyses confirmed that the ratio of 1:2 in this work is generally reasonable, although it is probably not the optimal choice.

10.1128/mSystems.00960-20.4 TABLE S1 The performance of the independent test set based on models trained by four different ratios of positives to negatives. Download Table S1, DOCX file, 0.03 MB. Copyright © 2020 Fu et al. 2020 Fu et al. This content is distributed under the terms of the Creative Commons Attribution 4.0 International license.

As we know, it is a challenging task to choose high-quality negative samples in network-based gene discovery with supervised learning. For instance, one limitation of our original negative sample construction is that some unknown HDFs are inevitably contained in the randomly selected negative samples and introduce noise to model training. To address this issue, we further examined the performance and biases of choosing different negative samples, including disease-associated genes (DAGs), HDFs from other viruses, essential genes, and genes with similar network degrees or expression levels to HDFs (see Materials and Methods for more details about the different negative data set preparations). Similar to our original model using randomly selected genes as negative samples, all of these new models were also trained by using RF with a 1:2 ratio of positives to negatives, and the corresponding performance is listed in Table 2.

**TABLE 2:** The performance of models based on different negative data set constructions

Negative data set construction	5-fold cross-validation^*a*^		Independent test^*a*^	
	AUC	AUPRC	AUC	AUPRC
Randomly selected genes	0.751 ± 0.003	0.554 ± 0.010	0.703 ± 0.013	0.483 ± 0.011
DAGs	0.662 ± 0.007	0.494 ± 0.011	0.552 ± 0.009	0.405 ± 0.011
HDFs from other viruses	0.625 ± 0.005	0.435 ± 0.008	0.539 ± 0.005	0.372 ± 0.005
Essential genes	0.703 ± 0.003	0.554 ± 0.010	0.762 ± 0.005	0.654 ± 0.012
Genes with similar T cell expression levels to HDFs	0.650 ± 0.002	0.461 ± 0.006	0.626 ± 0.009	0.441 ± 0.013
Genes with similar network degrees as HDFs	0.584 ± 0.003	0.399 ± 0.002	0.597 ± 0.002	0.401 ± 0.002

a The measurements are based on five different repeats of negative sample selections, which are reported as average ± SD.

Regarding choosing DAGs as non-HDFs, the levels of performance of the 5-fold cross-validation (AUC = 0.662 and AUPRC = 0.494) and the independent test (AUC = 0.552 and AUPRC = 0.405) are reasonably decreased in comparison to the performance of the original model. Biologically, HIV-1 HDFs tend to be DAGs. Of the known 1,144 HDFs used in our work and the initially collected 3,855 DAGs, 272 genes overlap (hypergeometric test, *P* = 4.44 × 10^−16^). In the context of GIANT, moreover, HDFs also share similar network topology properties with DAGs to a certain extent, which is exemplified in the corresponding box plots of network degree distributions (see Fig. S1 in the supplemental material). Since GIANT is a weighted network, note that all the reported network parameters in this work are also weighted, if not specified. Thus, choosing DAGs as negative samples increased the prediction difficulty. With respect to choosing HDFs from other viruses as negative samples, the levels of performance on the 5-fold cross-validation (AUC = 0.625 and AUPRC = 0.435) and independent test (AUC= 0.539 and AUPRC = 0.372) are considerably decreased in comparison to those in the original model. Again, these results may reflect the commonality of HDFs from different viruses. For instance, 108 out of the collected 834 influenza A virus subtype H1N1 HDFs overlap known HIV-1 HDFs (hypergeometric test, *P* = 5.05 × 10^−24^). Moreover, the commonality of HIV-1 HDFs and other viral HDFs is also reflected in their network properties (Fig. S1).

10.1128/mSystems.00960-20.1 FIG S1 The network degree distributions of HDFs and non-HDFs based on different selection methods. The 868 HDFs in the training set were used to calculate the degree distribution. In each non-HDF construction method, only one negative set was used to infer the degree distribution, although the negative set was repeatedly generated five times. The diamond symbol stands for the average value. The average network degrees for HDFs and six different non-HDF data sets along the *x* axis are 48, 36, 35, 42, 65, 50, and 41, respectively. Download FIG S1, TIF file, 2.0 MB. Copyright © 2020 Fu et al. 2020 Fu et al. This content is distributed under the terms of the Creative Commons Attribution 4.0 International license.

Regarding the model using essential genes as non-HDFs, the performance on the 5-fold cross-validation and independent test is fully comparable to that of our original model (Table 2). As we know, the essential genes perform important functional roles in human cells and often occupy unique network positions in gene networks (29, 30). For instance, the average network degree of essential genes is much higher than that of known HDFs (Fig. S1). In this context, the essential genes are not suitable for being selected as non-HDFs, although they have less chance to be HDFs. Indeed, when we conducted genome-wide HDF identification through the model using essential genes as negatives, 11,418 out of 25,085 human genes were predicted as HDFs when the FPR was controlled at 5%, implying biased results have been yielded from the new model. (Note that the prediction threshold corresponding to a 5% FPR was estimated from the model with a 1:2 ratio of positives to negatives.) Considering the network property differences between HDFs and essential genes, the majority of human genes tend to have comparatively more similar network features with HDFs rather than essential genes, and thus more human genes are prone to be predicted as HDFs.

When we further selected non-HDFs with similar network degrees to HDFs in the GIANT network, the model performance was also dramatically decreased, as expected (Table 2). Regarding the negative data set with similar expression levels to HDFs, the overall performance was also much lower than that of the original model (Table 2). The decreasing performance may be ascribed to the fact that the newly selected non-HDFs may still share similar network properties with HDFs (Fig. S1).

Based on the above computational experiments regarding the different negative sample constructions, we can conclude that using random proteins other than known HDFs as negative samples is still a reasonable choice, since the network properties of random genes can generally reflect the diversity of non-HDFs. As a network-based gene discovery method, moreover, the prediction specificity of the proposed method is also limited to the network properties of query proteins in the context of the GIANT network. For instance, other proteins with similar network properties to HDFs may have a high chance to be predicted as HDFs. We hope these pros and cons of negative sample constructions will be taken into consideration when developing new HDF prediction methods in the future.

### Comparison of the proposed method with an existing prediction method.

To our best knowledge, the method of Murali et al. is probably the only existing bioinformatics method to predict HIV-1 HDFs. Therefore, it is interesting and necessary to compare our method against Murali et al.’s method. In Murali et al.’s method, 908 positive genes and 455 negative genes were used to train and test models with 10 independent runs of 2-fold cross-validation. As a network-based prediction, their prediction was based on a human PPI network consisting of 71,461 interactions and 9,595 proteins. The adopted SinkSource algorithm was analogous to the functional flow algorithm, which was originally developed for protein function prediction. By following the method description of SinkSource in reference 16, we have implemented it through an in-house Python script. To ensure a fair performance comparison, we used the GIANT network, the training set, and the independent test set in our work to infer and evaluate the SinkSource-based prediction model. We compared the SinkSource-based model and our RF model through the 5-fold cross-validation and independent test. In general, the SinkSource-based model yielded a performance inferior to our RF model in terms of either AUC or AUPRC (see Fig. S2 in the supplemental material). For instance, the SinkSource-based model yielded an AUC of 0.654 and an AUPRC of 0.441 in the 5-fold cross-validation, while the corresponding values for our RF model were 0.751 and 0.554.

10.1128/mSystems.00960-20.2 FIG S2 Performance comparison of the SinkSource model and our predictive model based on the GIANT network. (A) ROC curves of the 5-fold cross-validation. (B) ROC curves of the independent test. (C) PR curves of the 5-fold cross-validation. (D) PR curves of the independent test. Parameters in parentheses of panels A and B denote the AUC values of different models, while parameters in parentheses of panels C and D stand for the AUPRC values. Note that the AUC/AUPRC values are reported as average ± SD. Download FIG S2, TIF file, 1.4 MB. Copyright © 2020 Fu et al. 2020 Fu et al. This content is distributed under the terms of the Creative Commons Attribution 4.0 International license.

To complement the aforementioned performance comparison, we also attempted to retrain our model on the basis of the training data set used in Murali et al.’s method. To this end, we first compiled a training set containing 868 HDFs (positive samples) and 434 human essential genes (negative samples). Note that the newly compiled training set is slightly different from the original training set of Murali et al., since some genes in Murali et al.’s data set did not occur in the GIANT network. Then, we retrained the predictive model based on the GIANT network encoding scheme and assessed the performance through the same 10 independent runs of 2-fold cross-validation. Finally, we compared the corresponding AUC values to roughly assess these two methods. Again, our method (AUC = 0.737 and AUPRC = 0.859) achieved better performance than Murali et al.’s method (AUC = 0.658 and AUPRC = 0.732, which were retrieved from reference 16), further suggesting that the GIANT network-informed HDF discovery is very competitive in comparison to Murali et al.’s method.

Murali et al.’s method and our method can be classified into two different types of network-based gene classification. As reported by Liu et al. (25), Murali et al.’s method belongs to a class of methods referred to as “label propagation,” while our RF-based method belongs to another class of methods called “supervised learning.” Although “supervised learning” is applied far less frequently than “label propagation” for network-based gene discovery (25), we have clearly demonstrated the promising performance of the proposed RF model in predicting HDFs. Apart from the methodological difference, it is also worth mentioning the different choices of negative samples in these two methods. Our method used random genes that are not HDFs as negative samples, while Murali et al. used essential genes as negative samples. Although most of the essential genes are unlikely to be HDFs, the unique network properties of essential genes may generate model bias. As discussed in the previous section, the randomly selected negative samples seem to be more suitable in developing the proposed RF-based predictive model.

### Important features contributing to the prediction of HDFs.

**FIG 3:**
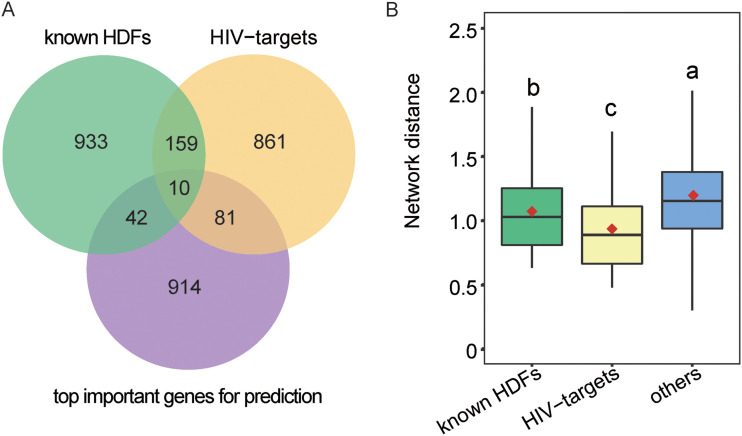
The relationships among top important genes for prediction, known HDFs, and HIV targets. (A) Venn diagram showing the overlaps among top important genes for prediction, known HDFs, and HIV targets. (B) Box plots showing the network distance between top important genes for prediction and known HDFs/HIV targets. For comparison, 2,000 human proteins other than known HDFs or HIV targets were randomly selected and compiled as a data set called “others.” Different lowercase letters indicate significant differences (*P* <  0.05), which were determined by one-tailed Wilcoxon rank sum test.

In general, the GIANT-based encoding scheme is of high dimensionality (i.e., 25,825 dimensions). In order to obtain a more optimized feature vector subset, the feature selection algorithm adopted in RF (i.e., the Gini algorithm) was conducted to reduce the dimensions to 1,047 (see Fig. S3 in the supplemental material). With these 1,047 top-ranked features, the corresponding RF model yielded an AUC value of 0.744 in the 5-fold cross-validation, which is very close to the performance based on the original GIANT-based encodings (AUC = 0.751). Although the feature selection did not result in performance improvement, it has rendered the model more concise and has allowed us to investigate the important features contributing to the prediction. The overlaps among the 1,047 genes corresponding to these 1,047 features (i.e., the top important genes for prediction), known HDFs, and HIV targets are shown in Fig. 3A. Interestingly, the top important genes for prediction significantly overlap HIV targets (Fig. 3A; hypergeometric test, *P* = 1.44 × 10^−10^). We further examined the top important genes in the context of GIANT network. The results showed these top important genes tend to be significantly closer to known HDFs/HIV targets in comparison to other human proteins (Fig. 3B; Wilcoxon test, *P* = 7.05 × 10^−8^ and *P* < 2.2 × 10^−16^, respectively). Collectively, these top important genes for prediction tend to be known HIV targets or neighbors of known HDFs/HIV targets, which may partly explain why the GIANT network is informative in distinguishing HDFs from non-HDFs.

10.1128/mSystems.00960-20.3 FIG S3 The performance of the 5-fold cross-validation based on different numbers of features. We first ranked the 25,825 features according to the corresponding Gini importance scores and further systematically investigated the performance based on the top-ranked features ranging from 1 to 25,825 with different steps. Briefly, the step was set to 1 when the number of features was in the range of 1 to 10: the steps were set to 10, 100, and 1,000 in the ranges of 10 to 100, 100 to 1,000, and 1,000 to 25,825, respectively. In general, the feature selection did not result in performance improvement, but the overall performance in terms of AUC was very close to the final performance when the top 1,000 features were selected. Moreover, we found that the Gini importance scores of the features ranked from 997 to 1,047 were the same. Thus, we chose the top 1,047 features as an important feature set for further analysis. Download FIG S3, TIF file, 2.1 MB. Copyright © 2020 Fu et al. 2020 Fu et al. This content is distributed under the terms of the Creative Commons Attribution 4.0 International license.

### Genome-wide screening of HDFs.

We used the proposed method to conduct genome-wide HDF screening. In brief, we used the corresponding five predictive models established by the 5-fold cross-validation to screen potential HDFs in the human genome. For each human protein, the final predicted score was averaged over the corresponding prediction scores from the five predictive models. Based on the final prediction scores, we ranked the 24,681 genes in the human genome, except 1,144 known HDFs. When the false-positive rate (FPR) was controlled at 5%, 857 HDF candidates were predicted. Note that the threshold corresponding to 5% FPR was estimated from the 5-fold cross-validation on the training set (ratio of positives to negatives = 1:2). In order to understand the characteristics of HDFs more comprehensively, we merged the predicted 857 HDF candidates and experimentally determined 1,144 HDFs into a data set containing 2,001 HDFs (see Data Set S1, sheet 1, in the supplemental material), which were collectively referred as HDF candidates in the subsequent analysis. It is worth noting that 423 out of these 2,001 HDF candidates are known HIV-targets, which is in line with previous observations that HDFs and HIV targets are strongly intertwined (16, 17).

10.1128/mSystems.00960-20.7 DATA SET S1 Sheet 1, list of the 2,001 HDFs. Sheet 2, list of complexes enriched with the 2,001 HDFs. Sheet 3, list of complexes enriched with 1,144 experimentally known HDFs. Sheet 4, the five groups of training and independent sets used in this work. Download Data Set S1, XLSX file, 0.3 MB. Copyright © 2020 Fu et al. 2020 Fu et al. This content is distributed under the terms of the Creative Commons Attribution 4.0 International license.

### Network analysis of experimentally validated and predicted HDFs.

**FIG 4:**
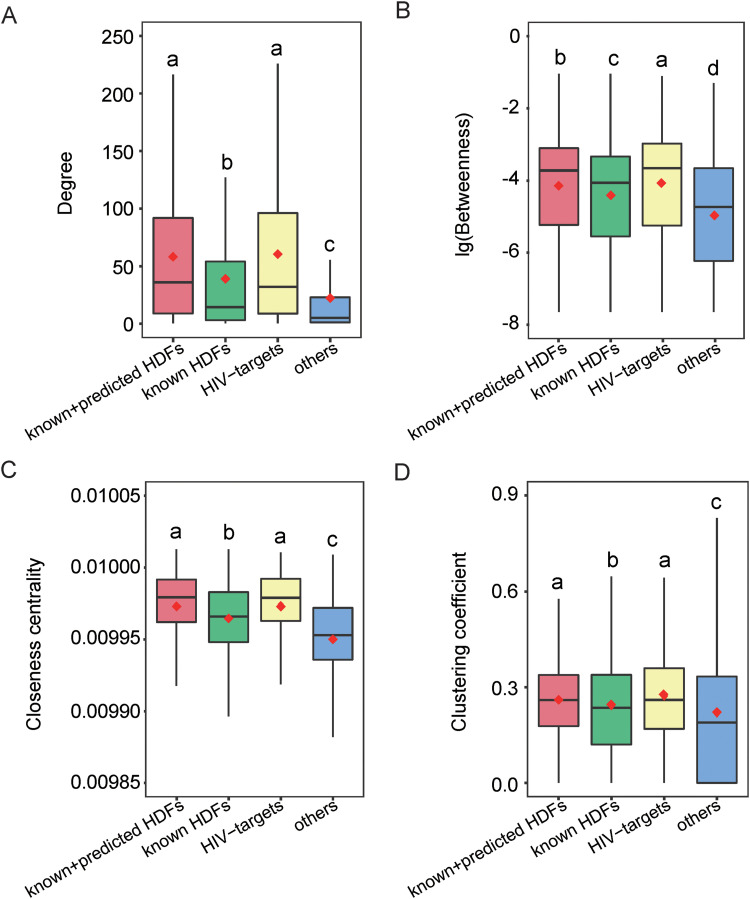
Comparison of topological parameters among HDF candidates (i.e., predicted and known HDFs), known HDFs, HIV targets, and other human proteins. Note that “others” denotes 2,000 randomly selected human proteins other than known HDFs or HIV targets. Panels A to D show the distributions of degree, betweenness, closeness, and the clustering coefficient, respectively. The red diamond stands for the average value. Different lowercase letters indicate significant differences (*P* < 0.05) determined by one-tailed Wilcoxon rank sum test.

To understand the network patterns of HDFs at a larger scale, we conducted network topology analyses of these 2,001 HDFs in the context of the GIANT network. We measured each HDF’s degree, betweenness, closeness centrality, and clustering coefficient in the GIANT network (Fig. 4). In brief, each gene in the network was regarded as a node and the edge was defined in case two genes are interacting. The degree of a gene denotes the number of the edges adjacent to the gene. The betweenness of a gene is defined as the proportion of the shortest paths between the interacting gene pairs that go through the node of interest. The closeness centrality of a gene is defined by the inverse of the average length of the shortest paths to all the other genes in the network. The clustering coefficient of a gene measures its local clustering within the GIANT network, which is defined as the number of existing edges between its neighboring genes divided by the maximal number of possible edges between its neighboring genes. Compared with other human genes, these 2,001 HDFs have significantly higher indicators in terms of degree, betweenness, closeness centrality, and clustering coefficient (Fig. 4; Wilcoxon test, all *P* values are <2.20 × 10^−16^). These network patterns indicated that HDFs are more likely to be hubs, bottlenecks, and centrally located in the GIANT network, which are very important to perform their functional roles. For instance, HDFs can control the information flow between nodes since they have many interacting partners and are located in the shortest paths between any two genes, which can probably explain why HDFs can help viruses effectively infect the host from the perspective of network biology.

For the purpose of comparison, we also calculated the corresponding network property distributions for experimentally known HDFs and HIV targets. Briefly, the experimentally known HDFs revealed significantly different network properties with other proteins or HIV targets (Fig. 4; Wilcoxon test, all *P* values are <2.20 × 10^−16^). When the predicted HDFs were taken into account, the predicted and known HDFs (i.e., the 2,001 HDFs) tended to have similar results for network degree, closeness centrality, and clustering coefficient with HIV targets (Fig. 4). Compared with Murali et al.’s work, the current network analysis further quantified the network property difference between HDFs and HIV targets. For instance, the 2,001 HDFs still reveal a significantly lower betweenness in comparison to HIV targets (Fig. 4B; Wilcoxon test, *P* = 0.0227), indicating that betweenness may serve as a potential indicator to further distinguish HDFs and HIV targets.

### Functional analysis of HDFs in the context of human complexes.

**FIG 5:**
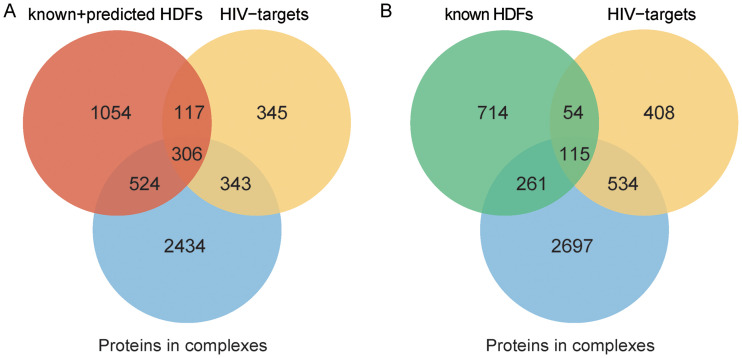
Venn diagrams showing the overlaps among HDFs, proteins in complexes, and HIV targets. (A) HDF candidates cover predicted and known HDFs. (B) HDFs only account for known HDFs.

Proteins are usually assembled into complexes and act as molecular machines to perform their functional roles (31). A protein complex contains multiple functionally diversified proteins (subunits). Previous studies have shown that viruses regulate the biological processes of host cells by manipulating host protein complexes (1, 7, 32). To conduct a large-scale investigation of HDFs in the context of human complexes, we collected all human protein complexes from a database of mammalian protein complexes called CORUM (33) and calculated the intersection of all HDF candidates and all proteins participating in complexes. The results indicated that the intersection is significant (hypergeometric test, *P* = 7.20 × 10^−223^), indicating that protein complexes are more likely to contain HDFs than randomly selected proteins (Fig. 5A). The preference for HDFs allows viruses to be more efficient in manipulating the corresponding complexes. Note that the experimentally known HDFs were also observed to significantly overlap proteins participating in complexes (Fig. 5B; hypergeometric test, *P* = 1.23 × 10^−62^).

Moreover, Fisher’s exact test was used to calculate the significance of complexes enriched with HDF candidates. The inferred *P* values were further corrected to *q* values (false-discovery rate) by the Benjamini and Hochberg method (34). In total, 585 of 2,824 complexes were observed to be significantly enriched with HDFs (*q* < 0.05). It is worth mentioning that 348 of these 585 complexes are also enriched with HIV targets (Data Set S1, sheet 2), further suggesting that HDFs and HIV targets are intertwined. The top 20 complexes enriched with HDFs are listed in Table 3, and all of them are enriched with HIV targets as well. For comparison, we only detected 53 complexes enriched with experimentally known HDFs (Data Set S1, sheet 3), which is far less than the number of complexes enriched with the 2,001 HDF candidates. It is worth mentioning that the number of enriched small complexes (i.e., those with ≤5 subunit members) was dramatically decreased when only taking the experimentally known HDFs into account. For instance, 348 small complexes were enriched with the 2,001 HDFs, while the number is only 10 for the experimentally known HDFs. We hope the incorporation of newly predicted HDFs can allow us to catch the relationship between HDFs and host complexes more comprehensively.

**TABLE 3:** Top 20 protein complexes enriched with HDFs

Complex ID	Complex name	No. of proteins	No. of HDFs	*q* value	No. of HIV targets
351	Spliceosome	143	81	2.50 × 10^−49^	52
1181	C complex spliceosome	80	46	4.62 × 10^−28^	35
193	PA700-20S-PA28 complex	36	28	2.13 × 10^−22^	20
181	26S proteasome	22	18	7.46 × 10^−15^	18
2825	BRCA1-RNA polymerase II complex	26	19	3.29 × 10^−14^	22
103	RNA polymerase II holoenzyme complex	24	18	7.88 × 10^−14^	22
2685	RNA polymerase II (RNAPII)	17	14	1.23 × 10^−11^	16
2755	17S U2 snRNP	33	19	1.29 × 10^−11^	15
32	PA700 complex	20	15	1.29 × 10^−11^	7
1332	Large Drosha complex	20	15	1.29 × 10^−11^	14
194	PA28gamma-20S proteasome	15	13	1.61 × 10^−11^	14
1183	CDC5L complex	30	18	1.61 × 10^−11^	14
2686	BRCA1-core RNA polymerase II complex	13	12	2.47 × 10^−11^	12
192	PA28-20S proteasome	16	13	6.56 × 10^−11^	13
191	20S proteasome	14	12	1.39 × 10^−10^	13
104	RNA polymerase II core complex	12	11	2.40 × 10^−10^	12
1335	SNW1 complex	18	12	1.87 × 10^−8^	12
728	CSA-POLIIa complex	13	10	5.72 × 10^−8^	11
3040	Multisynthetase complex	11	9	1.45 × 10^−7^	11
726	DDB2 complex	12	9	4.90 × 10^−7^	11

Indeed, the majority of the top 20 complexes enriched with the 2,001 HDFs are consistent with previous observations regarding the functional roles of HDFs associated with HIV-1 infection, which are exemplified as follows. For instance, HDFs are significantly presented in the spliceosome complex (*q* = 2.50 × 10^−49^). Of the 143 proteins in the spliceosome complex, 81 are HDFs. This suggests that many HDFs regulate viral infection by participating in mRNA splicing, which allows HIV-1 to prevent host downstream immune responses by inhibiting the production of the spliceosome. The proteasome is an important component of the ATP-dependent proteolytic pathway and regulates the degradation of most cellular proteins. It has been common knowledge that the proteasome is involved in HIV-1 replication. The proteasome is required for the release and maturation of infectious HIV-1 particles (35). Thus, HDFs were observed to be enriched in several proteasome-related complexes. SNW1 is a highly conserved protein complex associated with splicing and transcription. SNW1 is recruited by HIV-1 Tat to Tat:P-TEFb:TAR RNA complexes and is involved in Tat transcription by recruitment of MYC, MEN1, and TRRAP to the HIV-1 Tat-activated long terminal repeat (LTR) promoter, thereby overcoming the suppression of transcription elongation by negative elongation factors and stimulating transcriptional replication (36). Consistent with Kӧnig et al.’s work, we observed the SNW1 complex is enriched with HDFs.

In addition, we also discovered some complexes whose associations with HIV-1 HDFs had been rarely reported. For instance, RNA polymerase II catalyzes the transcription of DNA to synthesize mRNA. Obviously, HDFs regulate transcription to be primarily involved in the maintenance of viral latency, which is a crucial step in the life cycle of HIV-1. In the large Drosha complex, 15 of the 20 proteins are HDFs (*q* = 1.29 × 10^−11^), in which 11 HDFs are newly predicted and 4 HDFs are known HDFs. Drosha, a nuclease of the RNase III family, executes the initiation step of microRNA (miRNA) processing in the nucleus as the core nuclease, which can cleave primary miRNAs (pri-miRNAs) to release pre-miRNAs. Pre-miRNAs are processed into mature miRNAs, which play a role in regulating HIV-1 replication and infection (37–39).

Note that Murali et al. also examined the functionality of HDFs by seeking the locations of HDFs in the clusters of the human PPI network through a network graph clustering algorithm. Although the analysis strategy is different from ours, both studies share the same motivation of understanding the functional roles of HDFs from complexes or network clusters. Interestingly, some common clusters or complexes were identified. For instance, the identified spliceosome and proteasome complexes in our work are also related to the top 10 clusters highly connected with HDFs, as reported in Murali et al.’s work. Taken together, the aforementioned functional analysis of HDFs in the context of human complexes not only recapitulates known biology regarding human-HIV-1 interaction but also provides some hints to interrogate the functional roles of HDFs as well as the associated human complexes.

**FIG 6:**
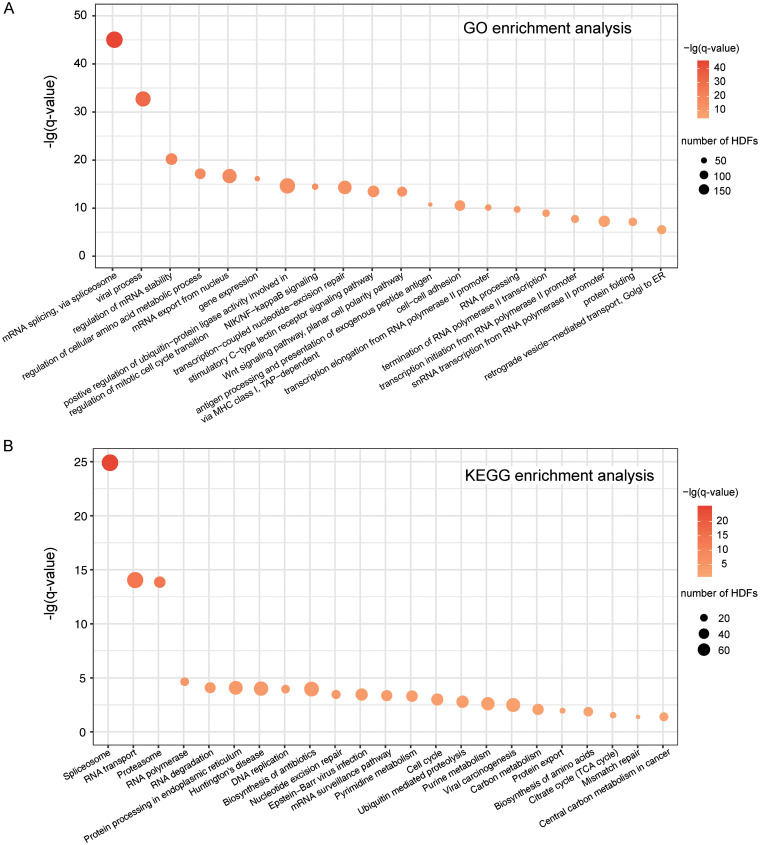
Functional enrichment analyses of the 2,001 HDF candidates. (A) GO enrichment analysis. (B) KEGG pathway enrichment analysis.

To complement the complex-based functional analysis of HDFs, we used DAVID (40) to perform Gene Ontology (GO) and Kyoto Encyclopedia of Genes and Genomes (KEGG) pathway enrichment analyses on the 2,001 HDF candidates. Here, we only took the GO category of biological process into account. REVIGO (41) was further employed to remove the redundancy of enriched GO terms. Likewise, a *P* value inferred from Fisher's exact test was further corrected to the *q* value by the Benjamini and Hochberg method (30). Thus, a total number of 41 GO terms were enriched (*q* < 0.05 [the complete GO terms are available in Table S2 in the supplemental material]), the top 20 of which are displayed in Fig. 6A. Similarly, 23 enriched KEGG pathways of the 2,001 HDF candidates were also inferred (*q* value of <0.05 [Fig. 6B]). In general, the GO/KEGG enrichment analysis has allowed us to understand the biological functions of HDFs more completely. For instance, we observed that HDFs are heavily associated with the spliceosome, proteasome, RNA polymerase II, and cell cycle through the functional annotations of GO/KEGG (Fig. 6A and B), which are consistent with previous functional analysis of enriched complexes to a large extent.

10.1128/mSystems.00960-20.5 TABLE S2 The 41 enriched GO terms in the 2,001 HDFs. Download Table S2, DOCX file, 0.03 MB. Copyright © 2020 Fu et al. 2020 Fu et al. This content is distributed under the terms of the Creative Commons Attribution 4.0 International license.

### Web server implementation.

To facilitate the research community, a simple web server called HDFP for managing and searching the 2,001 HIV-1 HDF candidates has been made freely accessible at http://zzdlab.com/HDFP. The web server was implemented with CentOS 7.4 and MySQL. It can display information about these 2,001 HDF candidates, including Entrez IDs, UniProt IDs, gene symbols, PubMed IDs, and prediction scores. Users can download all the detailed information regarding these 2,001 HDF candidates in an Excel or PDF format. Moreover, the source code of the proposed RF model, the training data set, and the independent test set used in this work are also downloadable through the web server.

### Conclusions.

In this work, we implemented an existing network-based gene discovery method to predict new HIV-1 HDF candidates from the GIANT network. The interaction scores of gene pairs in the GIANT network were used to construct the feature vectors of HDFs/non-HDFs. By applying the RF algorithm, we constructed an HDF predictor with reasonably good performance. Further comprehensive analyses on the combination set of experimentally determined HDFs and genome-wide predicted HDFs not only recapitulated the known knowledge regarding HIV-1 HDFs, but also provided further insights into the relationship between HDFs and HIV targets in the context of the GIANT network. In particular, HDFs revealed a significantly lower betweenness than HIV targets, although their network properties are generally similar when both experimental and predicted HDFs are taken into account. We further observed that the HDFs and HIV targets are highly intertwined, and they frequently co-occurred at the protein complex level, suggesting that this is an important avenue to decipher viral infection from the complexes enriched with HDFs. Taken together, our current results demonstrate that the GIANT network contains rich information regarding gene interactions and thus can be effectively employed for HDF identification. We hope the predicted HDF candidates can further guide hypothesis-driven experimental efforts to interrogate human–HIV-1 relationships.

## MATERIALS AND METHODS

### Data sets.

We collected 1,144 experimentally determined HIV-1 HDFs in total. A total of 868 of these 1,144 HDFs were compiled from three high-throughput HDF screening studies, including those by Brass et al. (13), König et al. (14), and Zhou et al. (15), which constituted the positive samples in our training data set. The remaining 276 known HDFs, collected through searching literature published from 2008 to 2017, were used to constitute the positive samples in the independent test set. Those human proteins other than known HDFs were randomly sampled as non-HDFs (negative samples). Considering the number of non-HDFs in the human genome should be much larger than that of HDFs, the ratio of positive to negative samples was set to 1:2 to build/assess a predictive model. Thus, we obtained a training data set containing 868 HDFs and 1,736 non-HDFs and an independent test set containing 276 HDFs and 552 non-HDFs. Since the negative data were much more available, we also repeated the selection of negative samples five times to investigate the robustness of model performance perturbed by the selection of negative samples. The full list of HDFs and non-HDFs in the five groups of training data sets and independent test sets is available in Data Set S1, sheet 4, or at http://zzdlab.com/HDFP.

For comparison, we also adopted five different ways to construct negative data sets. First, we used DAGs other than known HDFs as non-HDFs. To do so, we collected 3,855 DAGs from the OMIM database (https://omim.org/). After filtering out DAGs associated with known HDFs and HIV, 3,697 DAGs were retained, 2,288 of which were randomly selected as non-HDFs. Second, through literature searching we collected 5,506 HDFs from 11 other viruses, including influenza A virus subtype H1N1, human papillomavirus, dengue virus, hepatitis C virus, etc. After removing the same genes as HIV-1 HDFs, 5,365 HDFs from these 11 other viruses were retained and were randomly selected as negative samples. Third, we collected essential genes from three publications (42–44). After removing the redundancy, 2,290 essential genes were compiled, which were further used to construct negative samples. Moreover, proteins with a similar network degree to HDFs in the GIANT network were also selected as negative samples. For each HDF, we randomly selected two human proteins with similar degrees. By doing so, a negative data set was compiled, and the statistical test confirmed that the degree distributions between HDFs and the newly obtained negatives are similar (Kolmogorov-Smirnov test, *P* > 0.05). Finally, we also randomly chose genes with similar expression levels to HDFs to construct negative samples. To this end, we downloaded a set of T cell microarray data (accession no. GSE73968) (45) from the GEO database (https://www.ncbi.nlm.nih.gov/geo/). The expression level for each gene was further averaged by three replicates. For each HDF, two genes other than known HDFs but sharing similar expression values to the query HDF were randomly selected, and thus a negative gene set sharing similar expression levels to HDFs was obtained (Kolmogorov-Smirnov test, *P* > 0.05). Note that the different methods of non-HDF selections described above were also repeated five times to ensure the robustness of performance comparison.

Experimentally validated human–HIV-1 PPI data were collected from HPIDB 2.0 (46). After PPIs containing proteins without UniProt IDs were filtered, 1,638 human–HIV-1 PPIs between 1,142 human proteins and 19 HIV-1 proteins were obtained. We obtained 2,916 original protein complexes from CORUM (http://mips.helmholtz-muenchen.de/corum/) and filtered out complexes containing less than two subunits or complexes whose subunit members had unreviewed UniProt IDs. Thus, 2,824 complexes were retained. Experimentally determined human PPIs were collected from BioGRID (47), IntAct (48), and DIP (49). In total, 344,703 human PPIs covering 16,745 proteins were obtained to compile a human PPI network.

### GIANT encoding.

GIANT provides tissue-specific interaction maps, which can be downloaded from http://giant.princeton.edu/. In each tissue-specific network, the interaction probability for any gene pair is assigned. Considering the principal targets of HIV-1 are T cells, we used the T-cell-specific GIANT network to infer the feature vectors of HDFs and non-HDFs. For each HDF/non-HDF, the interaction probabilities with the 25,825 genes in the network were extracted to constitute the corresponding feature encoding. Thus, each HDF/non-HDF can be converted into a 25,825-dimensional feature vector.

### Machine learning algorithms.

In this work, we trained our predictive models through five commonly used machine learning algorithms (RF, SVM, LR, KNN, and NB), which were implemented in Python with the package scikit-learn (50). RF is an ensemble machine learning algorithm, which creates a forest of random uncorrelated decision trees to achieve the best possible result. SVM implements classification by mapping low-dimensional-input features into a high-dimensional space through a kernel function. LR is a generalized linear model, which constructs a regression model to estimate the probability of a binary classification by considering the relationships among multiple independent variables. The core idea of KNN is that if the majority of the *k* most neighboring genes in a feature space belong to a certain category, the query sample should also belong to this category. NB is a Bayes theorem-based algorithm with independent assumptions among input features. Here, we used Gaussian NB to allow training models with noninteger input features (51). We utilized MinMaxScaler in scikit-learn to conduct feature-wise standardization on the training data and applied the same transformation on the test set. In each algorithm, the most commonly used parameters were optimized through 5-fold cross-validation, while the other parameters were set as the default. More details about the parameter selection and optimization are available in Table S3 in the supplemental material.

10.1128/mSystems.00960-20.6 TABLE S3 Parameter selection and optimization in different algorithms. Download Table S3, DOCX file, 0.02 MB. Copyright © 2020 Fu et al. 2020 Fu et al. This content is distributed under the terms of the Creative Commons Attribution 4.0 International license.

### Performance assessment.

In this work, a 5-fold cross-validation and an independent test were employed to assess the predictive models. We used ROC curves to characterize the performance of our predictive model and further quantified the overall performance by the AUC value (52). In the meantime, the PR curve and the corresponding AUPRC value were also used to estimate the performance, which is commonly employed when the positive and negative samples are imbalanced. Briefly, an ROC curve plots a true-positive rate (TPR) against the FPR at different thresholds, whereas a PR curve plots precision values at different recall controls. The definitions of TPR (i.e., sensitivity or recall), FPR (i.e., 1 − specificity), and precision are as follows:

$$\text{TPR}=\text{sensitivity}=\text{recall}=\frac{\text{TP}}{\text{TP + FN}}$$

$$\text{FPR}=\frac{\text{FP}}{\text{TN + FP}}=1-\frac{\text{TN}}{\text{TN + FP}}=1-\text{specificity}$$

$$\text{precision}=\frac{\text{TP}}{\text{TP + FP}}\;$$

where TP, FP, TN, and FN denote the number of true-positive, false-positive, true-negative, and false-negative instances, respectively. In general, the closer the value of AUC/AUPRC is to 1, the more powerful the predictive performance is. All ROC/PR curves were generated by the ROCR package in R (53).

### Implementation of the SinkSource algorithm.

To compare our method with Murali et al.’s work, we implemented the SinkSource algorithm through a Python script by following the methodological details reported in references 16 and 54. Briefly, we represented the GIANT network as a weighted graph, *G =* (*V*, *E*), in which *V* denotes the set of nodes (i.e., genes) and *E* stands for the set of edges (i.e., interactions). We used $w_{\mathrm{uv}}$ to denote the weight of the edge (*u*, *v*) (i.e., the interaction probability for the edge in the GIANT network). We grouped *V* into three subsets, *V^P^*, *V^N^*, and *V*^0^. *V^P^* is the set of HDFs (positive samples), *V^N^* is the set of non-HDFs (negative samples), and *V*^0^ is the remaining set of nodes (unlabeled samples). For each node, $v\in V^{0}$, our task was to assess whether *v* should be classified as *V^P^* or *V^N^*. To address this issue, SinkSource constructed a function, *f*: *V*→[0, 1], where *f*(*v*) = 1 for each node in *V^P^*, *f*(*v*) = 0 for each node in *V^N^*, and *f*(*v*) for unlabeled nodes is “smooth” over *G* (16). Then, SinkSource assigned values for unlabeled nodes in *V*^0^ that minimize the function

$$S\left(G,f\right)=\underset{\left(u,v\right)\in E}{\sum }w_{\mathrm{uv}}{\left[f\left(u\right)-f\left(v\right)\right]}^{2}$$

given that the values of positive and negative nodes are fixed (16). The value of *f*(*v*) at each unlabeled node is defined as a weighted average of its neighboring nodes (16):

$$f\left(v\right)=\frac{\sum_{u\in N_{v}}w_{\mathrm{uv}}f\left(u\right)}{\sum_{u\in N_{v}}w_{\mathrm{uv}}}$$

where $N_{v}$ is the set of neighboring nodes of *v.* SinkSource used an iterative strategy to compute *f*(*v*). Let $f_{t}\left(v\right)$ be the value of node *v* at iteration step *t*. Note that $f_{0}\left(v\right)=0$ for each unlabeled node. We iteratively computed $f_{t}\left(v\right)$ for every unlabeled node until either it satisfied with

$$\sum_{v\in V^{0}}|f_{t}(v)-f_{t-1}(v)|\leq \;T$$

(*T *= 0.1 was set in our work) or 200 maximal iteration steps were reached. When the calculation terminated, the corresponding value of *f*(*v*) for each unlabeled node was obtained, and its label (*V^P^* or *V^N^*) can be further predicted.

We followed the strategy reported in reference 54 and removed edges with low interaction probability in the GIANT network to reduce the putative noise. Specially, an interaction probability of >0.15 was used to narrow down GIANT to a filtered network with 19,556 nodes and 3,052,895 edges.

### Calculation of network topological parameters.

The topological parameters (i.e., degree, network distance, betweenness, closeness centrality, and clustering coefficient) for genes in the GIANT networks were measured using the igraph package in R (55). Since GIANT is a fully connected weighted network, we only retained the top 0.1% of the edges after the network edge weight ranking for the convenience of network topology analysis. Thus, the GIANT network was converted into a network containing 333,452 edges. Regarding the calculations of degree and clustering coefficient, the interaction probability of each edge was assigned as the weight, whereas the parameter (1 − interaction probability) of each edge was assigned as the weight to infer network distance, betweenness, and closeness centrality.
