# Supplementary material for: Human Gene Functional Network-Informed Prediction of HIV-1 Host Dependency Factors
Source: mSystems. 2020 Nov 3;5(6):e00960-20. doi: 10.1128/mSystems.00960-20 (PMC7646529; doi:10.1128/mSystems.00960-20)
Supplement: TABLE S1 [file mSystems.00960-20-st001.docx]

**Table S1.** The performance of the independent test set based on models trained with four different ratios of positives to negatives.*^a^*

| *Ratio of positives to negatives in training* | AUROC | AUPRC |
| --- | --- | --- |
| 1:1 | 0.702±0.001 | 0.163±0.001 |
| 1:2 | 0.705±0.001 | 0.166±0.001 |
| 1:5 | 0.704±0.001 | 0.166±0.002 |
| 1:10 | 0.696±0.002 | 0.161±0.001 |

*^a^* Note that the ratio of positives to negatives in the independent test set was fixed at 1:10. The positive samples in the training set and independent set were the same as those used in training/testing our original models. We also repeated the selection of negative genes five times to investigate the robustness of model performance. Note that the results are reported in the form of “average±standard deviation”.
