# Supplementary figures and images for: Human Gene Functional Network-Informed Prediction of HIV-1 Host Dependency Factors
Source: mSystems. 2020 Nov 3;5(6):e00960-20. doi: 10.1128/mSystems.00960-20 (PMC7646529; doi:10.1128/mSystems.00960-20)

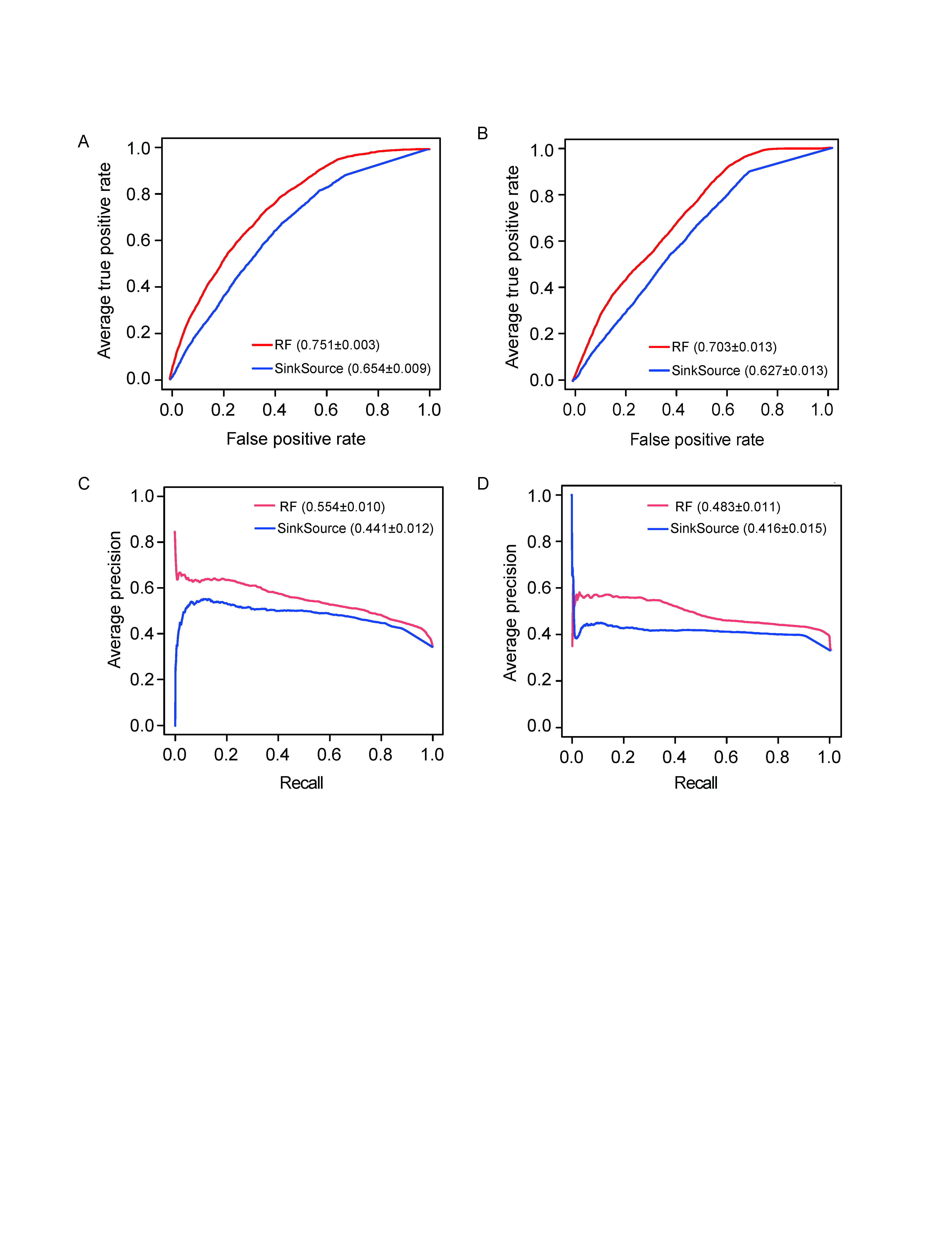

Supplement: FIG S2 [file mSystems.00960-20-sf002.tif]
