# Supplementary material for: Human Gene Functional Network-Informed Prediction of HIV-1 Host Dependency Factors
Source: mSystems. 2020 Nov 3;5(6):e00960-20. doi: 10.1128/mSystems.00960-20 (PMC7646529; doi:10.1128/mSystems.00960-20)
Supplement: TABLE S2 [file mSystems.00960-20-st002.docx]

**Table S2.** The 41 enriched GO terms in the 2,001 HDFs

| **Term_ID** | **Description** | **q-value** | **Count** |
| --- | --- | --- | --- |
| GO:0000398 | mRNA splicing, via spliceosome | 1.30×10^-45^ | 189 |
| GO:0016032 | viral process | 2.48×10^-33^ | 156 |
| GO:0043488 | regulation of mRNA stability | 7.60×10^-21^ | 89 |
| GO:0006521 | regulation of cellular amino acid metabolic process | 8.56×10^-18^ | 76 |
| GO:0006406 | mRNA export from nucleus | 2.79×10^-17^ | 138 |
| GO:0010467 | gene expression | 8.05×10^-17^ | 33 |
| GO:0051437 | positive regulation of ubiquitin-protein ligase activity involved in regulation of mitotic cell cycle transition | 3.07×10^-15^ | 162 |
| GO:0038061 | NIK/NF-kappaB signaling | 3.79×10^-15^ | 37 |
| GO:0006283 | transcription-coupled nucleotide-excision repair | 6.33×10^-15^ | 123 |
| GO:0002223 | stimulatory C-type lectin receptor signaling pathway | 4.04×10^-14^ | 89 |
| GO:0060071 | Wnt signaling pathway, planar cell polarity pathway | 4.39×10^-14^ | 66 |
| GO:0002479 | antigen processing and presentation of exogenous peptide antigen via MHC class I, TAP-dependent | 1.83×10^-14^ | 32 |
| GO:0098609 | cell-cell adhesion | 3.55×10^-11^ | 75 |
| GO:0006368 | transcription elongation from RNA polymerase II promoter | 8.06×10^-11^ | 37 |
| GO:0006396 | RNA processing | 2.07×10^-10^ | 39 |
| GO:0006369 | termination of RNA polymerase II transcription | 1.26×10^-9^ | 43 |
| GO:0006367 | transcription initiation from RNA polymerase II promoter | 2.06×10^-9^ | 47 |
| GO:0042795 | snRNA transcription from RNA polymerase II promoter | 6.62×10^-8^ | 85 |
| GO:0006457 | protein folding | 8.46×10^-8^ | 51 |
| GO:0006890 | retrograde vesicle-mediated transport, Golgi to ER | 3.55×10^-6^ | 57 |
| GO:0031047 | gene silencing by RNA | 9.00×10^-6^ | 34 |
| GO:0033209 | tumor necrosis factor-mediated signaling pathway | 1.31×10^-5^ | 35 |
| GO:0010501 | RNA secondary structure unwinding | 4.06×10^-5^ | 19 |
| GO:0010827 | regulation of glucose transport | 6.93×10^-5^ | 16 |
| GO:0032481 | positive regulation of type I interferon production | 9.82×10^-5^ | 20 |
| GO:1901796 | regulation of signal transduction by p53 class mediator | 1.27×10^-4^ | 95 |
| GO:0043044 | ATP-dependent chromatin remodeling | 1.33×10^-4^ | 13 |
| GO:0006366 | transcription from RNA polymerase II promoter | 1.35×10^-4^ | 202 |
| GO:0008543 | fibroblast growth factor receptor signaling pathway | 1.55×10^-4^ | 40 |
| GO:0006413 | translational initiation | 1.62×10^-4^ | 36 |
| GO:0050821 | protein stabilization | 3.66×10^-4^ | 35 |
| GO:0032508 | DNA duplex unwinding | 8.07×10^-4^ | 65 |
| GO:0006383 | transcription from RNA polymerase III promoter | 1.91×10^-3^ | 14 |
| GO:1904871 | positive regulation of protein localization to Cajal body | 2.94×10^-3^ | 8 |
| GO:1901998 | toxin transport | 4.73×10^-3^ | 14 |
| GO:0036498 | IRE1-mediated unfolded protein response | 9.51×10^-3^ | 18 |
| GO:0008283 | cell proliferation | 1.02×10^-2^ | 65 |
| GO:0035019 | somatic stem cell population maintenance | 1.05×10^-2^ | 19 |
| GO:0001649 | osteoblast differentiation | 2.05×10^-2^ | 25 |
| GO:0007005 | mitochondrion organization | 3.10×10^-2^ | 20 |
| GO:0043066 | negative regulation of apoptotic process | 4.20×10^-2^ | 84 |
