# Supplementary material for: Human Gene Functional Network-Informed Prediction of HIV-1 Host Dependency Factors
Source: mSystems. 2020 Nov 3;5(6):e00960-20. doi: 10.1128/mSystems.00960-20 (PMC7646529; doi:10.1128/mSystems.00960-20)
Supplement: TABLE S3 [file mSystems.00960-20-st003.docx]

**Table S3** Parameter selection and optimization of different algorithms.

| *Algorithms* | *Parameter selection and optimization* |
| --- | --- |
| RF | The optimal range of ‘n_estimators’ was set from 100 to 2000 with a step size of 100. Criterion was optimized between ‘entropy’ and ‘gini’. The final optimal parameters were 500 and ‘gini’, respectively. Other parameters were set as default. |
| NB | All parameters were set as default. |
| KNN | Euclidean distance was adopted. The range of the number of neighbors (*k*) was set from 1 to 10 with a step size of 1. The optimal *k* was 5. Other parameters were set as default. |
| SVM | The parameters *C* and *γ*  were optimized through grid search, where the ranges of *C* and *γ* were set to [2^-5^, 2^15^] and [2^-15^, 2^3^], respectively. Three kernels (‘linear’, ‘poly’, ‘rbf’) were also tested. Finally, *C* = 2, *γ* = 0.03125, and kernel = rbf were selected as the optimal setting. Other parameters were set as default. |
| LR | An L2-norm penalty function was assigned. *C* was chosen from four options (i.e. 0.0001, 1, 10, 1000), and the optimal *C* = 1. Other parameters were set as default. |
